# Clinical Applications of the Eosinophilic Esophagitis Diagnostic Panel

**DOI:** 10.3389/fmed.2017.00108

**Published:** 2017-07-14

**Authors:** Ting Wen, Marc E. Rothenberg

**Affiliations:** ^1^Division of Allergy and Immunology, Cincinnati Children’s Hospital Medical Center, Cincinnati, OH, United States

**Keywords:** eosinophilic esophagitis, reflux, EoE diagnostic panel, transcriptome, eosinophils, T helper type 2, histology, molecular profiling

## Abstract

Eosinophilic esophagitis (EoE) is a recently recognized upper gastrointestinal allergic disorder characterized by esophageal dysfunction (e.g., dysphagia) and esophageal eosinophilia of ≥15 eosinophils/high-power field in patients who have persistent esophagitis even on proton pump inhibitor (PPI) therapy. The histologic method is the gold standard of EoE diagnosis. However, EoE clinical symptoms do not always correlate with histology, and the histologic method has sensitivity and specificity issues due to the patchiness of EoE and the subjective nature of the method. The “EoE transcriptome” was initially discovered in 2006, which led to the invention of the EoE diagnostic panel (EDP). In addition to providing a definitive EoE diagnosis with high accuracy, the EDP has been useful in elucidating several key elements about the disease including the efficacy of specific drugs such as swallowed glucocorticoids and anti-IL-13 humanized antibody therapy, the relationship between EoE and PPI-responsive esophageal eosinophilia, and predicting the disease course and responsiveness to therapy. The EDP’s long-term potential arises from its plasticity to incorporate new genes and uncover novel disease pathogenesis. We expect that the EDP will be increasingly helpful for personalized medicine approaches and improved diagnostics and disease monitoring.

A molecular revolution swept through the fields of clinical diagnosis and predictive medicine at the turn of the century. Though advances in technology are affecting how basic research and clinical practice are performed, molecular diagnosis of diseases has been largely limited to cancer and genetic disorders. Conditions such as allergy have been underexplored in regard to the modern technical platform of molecular diagnosis.

Eosinophilic esophagitis (EoE) is a chronic, T helper type 2 (Th2)-associated immune disorder (1, 2) that involves food hypersensitivity. Typically, milk, egg, wheat, soy, corn, nuts, and fish represent the six food allergens that most commonly trigger disease activity in EoE (3). Diet elimination and steroid intervention are the two most effective therapies to quell disease activity and are frequently used together. At the cellular level, the inflammation is a well-concerted process orchestrated by local lymphocytes (primarily Th2), mast cells, and eosinophils within the esophagus and contributes to the clinical symptoms. Clinically, EoE is characterized by esophageal dysfunction (e.g., dysphagia) and is historically defined by tissue biopsy eosinophilia ≥15 eosinophils/high-power field (EOS/HPF), a cut-off agreed to by a panel of experts and referred to as a consensus recommendation (CR) (4, 5). Both histology and clinical symptoms, as well as an 8- to 12-week proton pump inhibitor (PPI) trial, have been historically required for diagnosis of EoE but it is now becoming apparent that PPI responsiveness does not necessarily differentiate esophageal eosinophilia into distinct clinical entities. Typically, as mentioned in the CR, five to six biopsy samples are required to reach a satisfactory sensitivity, with one biopsy yielding only ~55% sensitivity (4, 6).

Thus far, histologic examination is the only widely accepted EoE diagnostic test. Yet, the disadvantages of the conventional histologic method are its subjectivity, time requirement, reliance on experts, and expense. The histologic analysis of esophageal specimens can be confounded by other variables, including interobserver variation, size differences in microscopic HPF among multiple microscope manufacturers, and the patchiness of the disease (7). Moreover, the histologic detection of tissue eosinophilia is non-specific to EoE, as there are several other diseases sharing similar esophageal eosinophilia, and cannot identify specific exposure to medications (such as glucocorticoids) nor differentiate patients with EoE remission from patients who do not have EoE (both have no eosinophilia). Therefore, the only available “gold standard” method has limitations, which a next-generation diagnostic method could overcome.

The EoE transcriptome is 1610 genes dysregulated at differing magnitudes and bidirectionally in the esophagus and was identified by Blanchard et al. in 2006 (8) together with subsequent studies (9, 10). This transcriptome serves as the foundation for the molecular differentiation of EoE from other disorders, such as gastroesophageal reflux disease (GERD). With having the raw threshold cycle (Ct) values of quantitative PCR (qPCR), the critical next step was to develop a way to interpret the results in a way that general physicians and patients could comprehend and use. One of the unique features of the EoE diagnostic panel (EDP, US Patent pending 47108-510N01US) is the novel dual algorithm based on the raw qPCR Ct values (11). Briefly, the first algorithm is a clustering analysis based on the Pearson correlation of a 77-gene/dimension hypothetical space. With 50 upregulated genes and 27 downregulated genes, the bidirectional dysregulation provides a pronounced contrast for signature recognition. The dendrogram (hierarchical tree) is derived on the basis of the inter-sample distance metrics aided by commercial analysis softwares such as GeneSpring (Agilent Inc.). The first branch of the dendrogram tree serves as a diagnostic bifurcation point. The “EoE score” algorithm performed in parallel is essentially a mathematical summing-up of the relative Ct value change of each gene to the housekeeping gene (*GADPH*) considering the bidirectional changes (+ and − vectors). The end read-out of this algorithm is an absolute integer providing definitive EoE diagnosis and linearly correlated with the disease severity. This direct output allows the physicians to readily assess the disease status and to plan for corresponding therapies. It is worthwhile to mention that the unique qPCR array design combined with the practical scoring algorithm can be readily applied to the diagnosis of other allergy inflammatory diseases with minimal modifications, such as other eosinophilic gastrointestinal (GI) disorders (EGIDs) and atopic dermatitis (12).

Of note, the EDP’s compatibility with formalin-fixed, paraffin-embedded (FFPE) samples provides a valuable and unique opportunity to retrospectively study archived paraffin samples (11). Importantly, this crucial feature has been validated externally by at least two independent groups (13, 14). Whereas FFPE RNA is known to be highly degraded as a function of time, the EDP signature acquisition has been intentionally designed with short amplicons, so that it can be readily used with paraffin-archived samples (highly degraded/fragmented RNA) that are usually not compatible with RNA sequencing. One can imagine how many clinical questions can be answered with a vast storage of FFPE-archived pools and the ample amount of associated information regarding clinical outcome.

Importantly, since the original publication of the EDP by Wen et al. (11), multiple studies successfully reproduced the diagnostic merit at external institutions with independent samples, using the same molecular platform and array design. Notably, Dellon et al. performed a well-controlled EDP study at University of North Carolina using both FFPE and matching RNAlater™ samples (13). A total of 72 samples, representing paired FFPE and RNAlater™ specimens from 9 EoE cases and 3 GERD controls were analyzed by EDP (13). A robust correlation was demonstrated between paired FFPE and RNAlater™ samples. Moreover, by the reported EDP score, EoE was well distinguished from control GERD samples without overlap and with excellent diagnostic merit (13). The second external validation was led by Drs. Genta and Lash from Miraca Life Sciences (TX, USA), a large-scale GI disorder diagnostic corporation based in the USA (14). This study encompassed a relatively large cohort of 265 FFPE samples randomly selected from their sample archive, which repeatedly showed an equally excellent diagnostic merit of the EDP (11, 14). Collectively, these studies show that the EDP is a highly reliable and reproducible molecular procedure to distinguish EoE from normal and GERD tissues.

Besides providing definitive EoE diagnosis, the EDP’s clinical utility has been demonstrated in multiple registered clinical trials. In a double-blinded, placebo-controlled fluticasone clinical trial, the EDP was used as a molecular gauge to evaluate the treatment efficacy and remission status of EoE (15). Of note, the EDP-derived EoE score, a readily available and interpretable parameter, was directly used in this clinical trial to assess EoE molecular severity and intervention efficacy in a quantified fashion. It was also found that certain embedded genes were able to predict fluticasone responders vs. non-responders, suggesting a potential for predictive medicine in the field of Th2 allergic GI disorders (15). Likewise, in a recent anti-IL-13 humanized antibody clinical trial (QAX576, Novartis) (16), a similar transcriptome analysis was used to monitor EoE activity following this anti-Th2 cytokine intervention. Using transcriptome analysis, there was a remarkable reversibility of nearly all EoE-associated genes, including reduction in expression of the cardinal eosinophil chemoattractant (*CCL26*), mast cell signature genes (e.g., *CPA3*), and increased levels of the barrier gene *DSG1*. Notably, the molecular improvements were even more impressive than the histologic improvement, which did include reduced levels of esophageal eosinophilia and a trend for improvement in dysphagia. The improved molecular signature is likely providing early insight into the potential positive impact that anti-Th2 cytokine therapy may have in EoE.

The EDP as a molecular platform also represents a tool for identifying the patients whose disease is most likely to respond to anti-Th2 cytokine treatment (16). This personalized medicine approach is potentially applicable to not only the management of EoE but also other Th2 disorders, such as asthma. Anti-Th2 cytokine intervention (anti-IL-5) has been proven to be quite efficient in eosinophilic asthma and was recently approved by the FDA (17, 18). We envision that anti-IL-13 therapy, guided by the personalized assessment of EDP, will likely follow a similarly successful path to clinical utility.

The EDP is also useful in defining new disorders and disease subentities or in molecularly analyzing comorbidities, such as in defining the molecular signature of PPI-responsive esophageal eosinophilia (PPI-REE) (16). PPI-REE, a large dilemma and confounding factor in the field of EoE, was recently identified in patients with endoscopic and symptomatic features of EoE but who respond well to PPI mono-therapy (4, 19, 20). It remained unclear whether PPI-REE represented an independent clinical disorder or is a subentity of either EoE or GERD (20). The initial EDP study demonstrated that the signature of PPI-REE largely overlapped with that of EoE, suggesting that they share the same disease process and may represent a continuum (11). This is consistent with other studies using alternative methods showing PPI-REE and EoE are indistinguishable (20, 21). However, with the genome-wide screening between PPI-REE and EoE now being readily available, we hope that the next version of the EDP would have a component to prospectively distinguish PPI-REE and EoE before the PPI therapy, which could save significant clinical resources and improve the clinical care quality (e.g., reducing time to effective treatment). The EDP is not a fixed design in terms of molecular composition. With the same quantification algorithm and technical platform, any new leads that have potential in predictive and personalized medicine, as well as those genes helpful to solve diagnostic dilemmas, could be readily incorporated to enhance the value of this molecular panel.

As for the *bona fide* feasibility of distinguishing EoE and PPI-REE, it is now generally accepted that EoE and PPI-REE are very similar subentities and that PPI-REE may not have a pathogenesis initiated by GERD (11, 19). The PPI-REE transcriptome study (16), as well as a recent genome-wide transcriptome study (22), directly demonstrated an overlapping transcriptome between EoE and PPI-REE. There is only a marginal difference between the two entities, with a potassium channel (KCNJ2/Kir 2.1) potentially being differentially expressed. A collaborative effort is ongoing to validate the utility of this gene in EoE vs. PPI-REE differential diagnosis before PPI therapy. A genome-wide screening with a large cohort number and a sufficient sequencing depth will have the potential to identify some low-abundance differential genes, serving as the foundation for future diagnostic tests.

Another useful application of the EDP is deciphering disease endotypes. Recently, it has become increasingly clear that many human diseases are complex disorders made up of several clinical and pathologic variants with disparate underlying etiology. For example, in asthma, it is now appreciated that there are multiple endotypes including eosinophil-high, neutrophil-high, and Th2 cytokine-high subgroups. Unlike asthma, there is no such approach being explored in the field of human allergic GI disorders. Of note, there is a considerable amount of variability among the EoE cohort in terms of molecular scoring and expression pattern (11). It is conceivable that EoE also has different molecular manifestations due to distinct causes and responses, even though all samples meet the >15 EOS/HPF histologic diagnostic standard. By molecular profiling, it is hoped that a better understanding of EoE subtypes will promote a more personalized medicine approach to EoE management.

Distinguishing GERD and EoE can be clinically difficult due to several confounding factors, including symptom overlap, histologic similarity, and the presence of PPI-REE (4). Though several tests can be used for this differential diagnosis [e.g., impedance (23), pH probe (24), endoscopy (25), and histology (26)], the molecular diagnostic panel excels in distinguishing EoE and GERD. In the original EDP study (11), using FFPE samples, Wen et al. showed that the EDP is able to distinguish impedance/pH-selected patients with GERD from patients with EoE, as the transcriptome of the former is more similar to normal controls. Although most of the GERD [>98% (27)] cases do not have as great a degree of eosinophilia as EoE does (>15 EOS/HPF), the caveat herein was whether the EDP would distinguish EoE from atypical GERD samples with high eosinophilia. In this same study, Wen et al. showed a similar transcriptome between EoE and EoE + GERD comorbidity, suggesting that the EoE signature is dominant. Though there is little doubt that the EDP is able to distinguish EoE from GERD without a high eosinophil level, it remains unclear whether the EDP can dissect out the GERD components to differentiate GERD with high eosinophilia from GERD with comorbid EoE, as the high eosinophilia may be mimicking the dominant EoE signature. Notably, this type of differential diagnosis is of less clinical importance because it is more significant to distinguish GERD with low eosinophilia from EoE due to distinct management.

It is worthwhile to mention that human disease development and remission are usually a progressive and dynamic process rather than an abrupt “all or none” change. Many common disorders, such as diabetes and hypertension, have ambiguous areas between normal and diseased cutoffs, indicating a borderline state and equilibrium between promoting and inhibitory factors. Following food allergen exposure, EoE development is a linear course regulated by a series of counterbalanced components. Therefore, a “black and white” threshold may not truly exist. In this sense, any EoE score value around the 333 cutoff represents a “snapshot” of the equilibrium reached between pro-EoE factors (e.g., food allergen presence, Th2 cytokine production, mastocytosis, inflammatory cell proliferation) and anti-EoE factors (e.g., elemental diet, steroid exposure, anti-Th2 therapy). One can imagine that such an integer score can be used to monitor disease status and responses to therapy intervention. Indeed, the aforementioned Dellon et al. study demonstrated the key function of the EDP in assessing the efficacy of EoE clinical management (9), an external validation study with 265 independent samples. Therefore, multiple lines of evidence show that the EDP serves well as an EoE status monitoring tool with prognostic values, a personalized quantification that can barely be achieved by histologic analysis.

With the non-invasive trend sweeping through the diagnosis of other disorders, the field of EoE has an urgent need for a non-invasive or less invasive method to replace the biopsy-based examination (28). It is conceivable that the current molecular platform of the EDP, when combined with its associated algorithms, could be used for a non-invasive diagnosis of EoE. Burgeoning progress has been made regarding the circulating biomarkers for EoE, including miRs (29) and certain cytokine signaling molecules (30). In the next 5 years, it is also possible for the EDP to incorporate some genetic components from the results obtained from some promising non/less invasive platforms, such as oral cytobrush, esophageal string test (31), and swallowed sponge test (32), or even more biomarkers from the patients’ blood (30). The recently reported increase in levels of circulating eosinophil progenitors (33) in patients with EoE may render value in embedding certain eosinophil progenitor markers in the EDP, which could be potentially useful on biological samples not derived from esophageal biopsy. One of the primary goals of a future version of the EDP is to embed a non-invasive or less invasive component to facilitate improved diagnosis and monitoring and quality of life of patients with EoE.

The EoE transcriptome has been deposited online by the Rothenberg group for public use (Microarray 2006 GEO GSE8853; RNA sequencing 2014 GEO GSE58640). Utilizing modern bioinformatics is a key approach to identifying new information from old data to guide basic research. We propose several questions and unmet areas that we think are meaningful undertakings to better guide EoE translational/clinical research:

1. What is the overlap between the EoE transcriptome and those of other Th2 human disorders, including GI (EGIDs) and non-GI allergic disorders (asthma, atopic dermatitis)? Within the overlap, what functional elements are shared by EoE and other Th2 disorders?
2. Since the EoE miRome (small RNA transcriptome) was also elucidated by the Rothenberg group (29, 34), what is the functional network between the EoE transcriptome and the corresponding miRome considering the robust roles of miRs in regulating gene expression? Can the dysregulated miRome explain or regulate the dysregulated EoE transcriptome? If so, what is the miR–gene interaction network? Which is the cause, which is the consequence, or are both consequences of further upstream factors?
3. With more and more cell type-specific data deposited in GEO, are we able to perform a “deconvoluted” bioinformatic dissection (35) to determine which part of the EoE transcriptome is contributed by each cell type?
4. In the same line of thinking as question 3, this field urgently calls for a single-cell analysis to decipher the contribution of each cell type and their developmental relationship in parallel. The recently published Drop-seq technique seems to be well suited for this purpose (36).
5. With the EoE genome-wide association study (GWAS) data published by the Rothenberg group (37), it remains to be determined what percentage of the EoE transcriptome is regulated at the genomic DNA level. If EoE is a known multifactor genetic disorder, how could an isolated single-nucleotide polymorphism difference explain the vast dysregulated transcriptome in EoE? Are the mutations identified by the GWAS family specific? If yes, at what point do they converge? The causal links between the GWAS findings and EoE transcriptome need to be established.

In summary, the EDP and associated dual algorithm represent a cutting-edge approach poised to advance EoE and related inflammatory disorders to a 21st century molecular and precision medicine level (see Figure 1 summarizing the utilities of the EDP in studying the EoE signature). Moreover, the transcriptome study also offers strong potential for future personalized medicine practice with prognosis-predicating values. In the next 5 years, we predict that new components embedded in the EDP will distinguish PPI-REE from EoE, define EoE endotypes, and hopefully suggest the best treatment or predict the effectiveness of a certain regimen. This proposed achievement will be greatly facilitated by genome-wide sequencing (10) and genomic DNA variant studies such as GWAS and whole-exome sequencing (37). Though the EDP is the first to carry molecular signature interpretation from the field of cancer to that of digestive tract disorders, we also envision that the same success could be reproduced in the diagnosis, monitoring, and clinical management of other allergic inflammatory disorders, such as eosinophilic gastritis and eosinophilic colitis.

**Figure 1:**
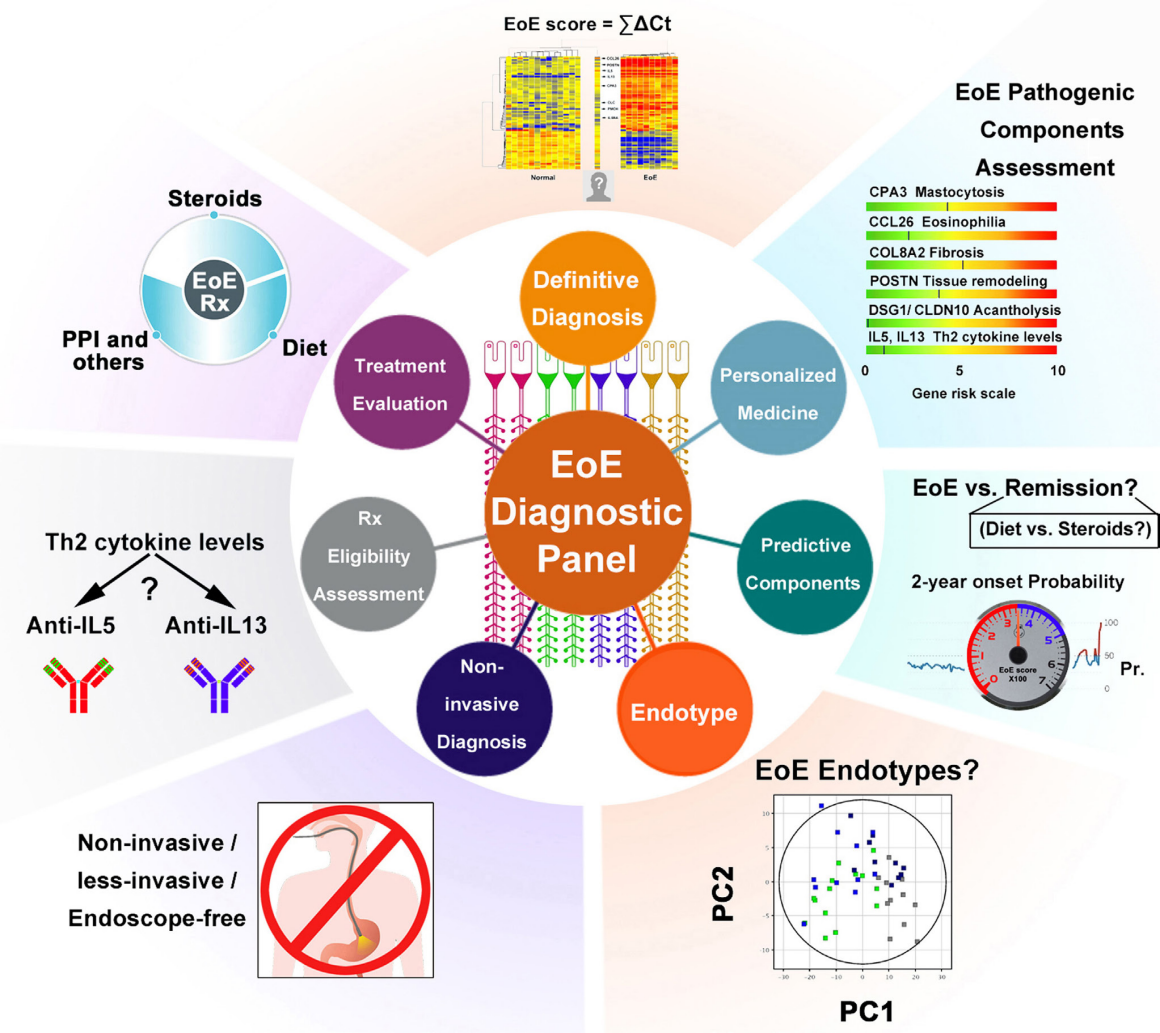
Schematic summary of the EDP’s multiple functions. Multiple components of the EDP are graphically summarized to illustrate the applicable functions of the molecular panel. Some are in clinical practice, whereas others are potentials for future diagnostic utilities. Abbreviations: PPI, proton pump inhibitor; Th2, T helper type 2; Rx, treatment; EoE, eosinophilic esophagitis; PC, principal component; ΣΔCT, EoE score = Σ (Ct. downregulated gene − Ct. GAPDH) − Σ (Ct. upregulated gene − Ct. GAPDH) (refer to Wen et al. study); Pr., probability; 2-year onset probability, the estimated possibility of EoE relapse for patients who currently have EoE in remission.

## Author Contributions

TW and MR collaboratively studied the topic and wrote the review together.

## Conflict of Interest Statement

TW and MR are coinventors of the EDP, a patent owned by CCHMC. There is no commercial interest to disclose at the moment.
